# AI/ML-Assisted Detection of *HMGA2* RNA Isoforms in Prostate Cancer Patient Tissue

**DOI:** 10.3390/ijms27010196

**Published:** 2025-12-24

**Authors:** Bor-Jang Hwang, Oluwatunmise Akinniyi, Sharon Harrison, Denise Gibbs, Charles Waihenya, Andrew Gachii, Precious E. Dike, Bethtrice Elliott, Fahmi Khalifa, Camille Ragin, Valerie Odero-Marah

**Affiliations:** 1 Department of Biology, Morgan State University, Baltimore, MD 21251, USA; bor-jang.hwang@morgan.edu (B.-J.H.); prdik1@morgan.edu (P.E.D.); bethtrice.elliott@morgan.edu (B.E.); 2 Department of Electrical and Computer Engineering, Morgan State University, Baltimore, MD 21251, USA; olaki58@morgan.edu (O.A.); fahmi.khalifa@morgan.edu (F.K.); 3 Cancer Prevention and Control Program, Fox Chase Cancer Center, Philadelphia, PA 19111, USA; sharon.harrison2@fccc.edu (S.H.); denise.gibbs@fccc.edu (D.G.); camille.ragin@fccc.edu (C.R.); 4 The African Caribbean Cancer Consortium, Philadelphia, PA 19111, USA; waihenyacharles@gmail.com (C.W.); andrewkgachii@gmail.com (A.G.); 5 Department of Surgery, University of Nairobi, Nairobi 00100, Kenya; 6 Department of Pathology, Aga Khan University, Nairobi 00100, Kenya

**Keywords:** RNA in situ hybridization quantification, isoform-specific gene expression, high mobility group AT-hook 2, AI, machine learning image analysis

## Abstract

RNA In Situ Hybridization (RISH) is a powerful tool for spatial gene expression analysis, yet its quantitative use remains limited by the high cost and inaccessibility of commercial software, particularly in under-resourced settings. This study developed an Artificial Intelligence/Machine Learning (AI/ML)-assisted RISH quantification pipeline to evaluate expression patterns of High Mobility Group AT Hook-2 (*HMGA2*) in prostate cancer (PCa), focusing on racial disparities. We created a machine learning model capable of analyzing RISH images. Expressions of full-length (wild-type) and truncated *HMGA2* isoforms were assessed in tissues from 85 men of African descent, European American, and Asian descent. A training dataset was generated for supervised learning analysis of the full cohort. RISH findings revealed that the wild-type *HMGA2* isoform was significantly more abundant in tumors from men of African descent and positively correlated with increasing Gleason grade. The truncated isoform was less abundant and did not display a consistent expression pattern across racial groups. These results demonstrate the feasibility of AI/ML-based RISH quantification and suggest that elevated wild-type *HMGA2* expression may represent a biomarker linked to prostate cancer aggressiveness and racial disparities. These findings highlight the importance of interdisciplinary collaboration and equitable computational tools in advancing biomarker discovery and addressing cancer health inequities.

## 1. Introduction

Prostate cancer (PCa) continues to be a major threat to men’s health worldwide, with a steady increase in incidence rates over the last 10 years [1]. In the United States, between 2014 and 2021, the incidence rate of PCa increased by approximately 3% per year [1]. African American men experience a higher incidence and worse clinical outcomes compared to other racial groups [2]. Several factors contribute to PCa progression, including oxidative stress, aberrant androgen receptor (AR) signaling, epithelial–mesenchymal transition (EMT), and dysregulated growth factor signaling pathways [3,4,5].

One biomarker implicated in PCa progression and drug resistance is High Mobility Group AT Hook-2 (*HMGA2*), which is associated with aggressive phenotypes and resistance to therapy [5,6,7,8]. Our recent publication identified *HMGA2* as a biomarker of enzalutamide resistance in PCa [8].There are several isoforms of HMGA2; however, the most studied are truncated HMGA2 and the full-length wild-type HMGA2 [9]. Truncated HMGA2 lacks the C-terminus acidic domain and the 3′ untranslated region (UTR), making it resistant to regulation by *let-7* microRNAs (miRNA), which normally suppress HMGA2 expression [9]. Alternative splicing produces the wild-type and truncated forms that share exons 1–3, but differ at the 3′ end. The wild-type *HMGA2* isoform includes exon 5 and a long 3′ untranslated region (L-3′UTR), whereas the truncated isoform replaces this region with exon 4 and a short 3′UTR (S-3′UTR) [9,10]. Recent findings indicate that while wild-type HMGA2 promotes EMT, truncated HMGA2 enhances cell proliferation, oxidative stress, and ferroptosis in PCa [7,10].

RNA In Situ Hybridization (RISH) is a hybridization method that enables the visualization and localization of the target mRNA transcript within cell and tissue samples. RISH uses sequence-specific probes to hybridize to the target mRNA sequence. Unlike traditional methods such as reverse transcription polymerase chain reaction (RT-PCR) or RNA sequencing, which only provide the abundance of the RNA of interest, RISH also reveals the spatial distribution of gene expression within tissue [11,12,13]. RISH enables the detection of gene expression at the single-cell level while preserving the spatial context of the tissue. Its importance has grown in cancer research, where tumor heterogeneity, cellular composition, and microenvironmental factors can influence gene expression and response to therapy. Technologies such as RNAscope have significantly improved the resolution and sensitivity of RISH by reducing the background noise ratio and analysis time [12]. However, current methods used for quantifying RISH signals are often limited by subjective interpretation and reliance on expensive software.

Machine learning (ML) is a branch of artificial intelligence (AI) in which computer systems learn intricate details, i.e., patterns and features, from data to make predictions or decisions [14]. In the context of RISH image analysis, deep-learning-based methods provide accurate cell segmentation and spot detection, enabling precise quantification of single-cell gene expression and streamlining the analysis of spatial transcriptomics data [15]. ML has become a transformative tool in biomedical research, particularly in image analysis, due to its ability to process large datasets and detect complex patterns that may not be readily apparent to the human eye. Recent studies have applied ML techniques to analyze RISH images for purposes such as quantifying gene expression, mapping spatial transcriptomics, and identifying biomarkers [16,17,18]. These approaches often rely on segmentation, where the ML model separates areas of interest (e.g., RNA dots) from the background. Segmentation is critical for accurate RNA quantification and for understanding spatial distributions within tissues [19]. Previous work has demonstrated that convolutional neural networks (CNNs), a widely used class of deep learning (DL) models for image processing, excel in segmenting biological structures, such as cells, nuclei, and RNA signals, from complex and noisy images [20].

In this study, we propose to apply AI/ML approaches to develop tools for quantifying *HMGA2* isoforms in PCa tissues using RISH, which will be superior to subjective scoring methods. Our objectives are to evaluate the spatial expression patterns of *HMGA2* isoforms, assess their association with clinical parameters such as tumor grade, metastatic potential, and therapy resistance, and determine whether expression patterns vary across racial groups.

## 2. Results

### 2.1. RISH Staining of a Cohort of PCa Patient Tissue and Subjective Analysis of HMGA2 Isoform

**Figure 1:**
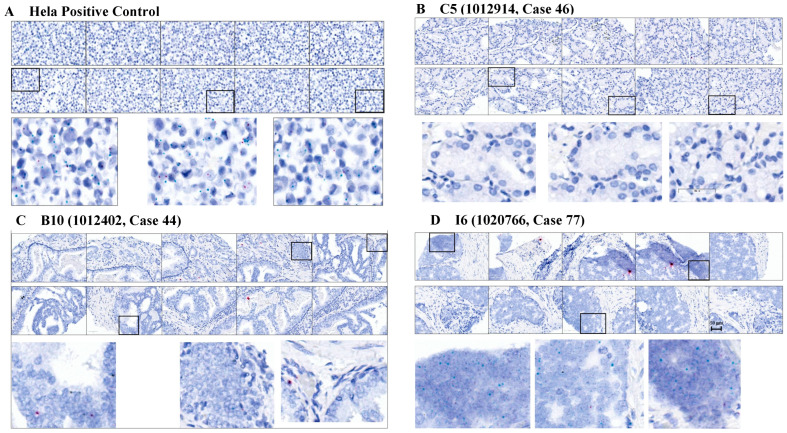
RISH images of tissue samples from Fox Chase tissue microarray: (**A**) HeLa cells served as a strong positive for both wild-type (blue dots) and truncated *HMGA2* (red dots), with strong signals on both; (**B**) sample C5 showed the weakest signals for both wild-type and truncated *HMGA2* mRNA; (**C**) sample B10 from a White patient with Grade III adenocarcinoma exhibited moderate signals for both wild-type and truncated *HMGA2*; (**D**) sample I6 from a Black patient with metastatic PCa expressed strong signals for wild-type *HMGA2* mRNA in patient tissue. Scale bar = 50 μm.

We initially performed RISH staining on a PCa patient tissue microarray containing 12 Black/African American and 27 White/European American tissue samples and present representative images in Figure 1. HeLa cells were used as a positive control, with a score of 5 assigned for both blue (wild-type) and red (truncated) dots (Figure 1A). In general, wild-type *HMGA2* expression was higher than truncated *HMGA2* expression across all groups, including HeLa cells. Therefore, the truncated score for HeLa remained 5, even though the number of red dots was lower than the blue dots. Patient tissue C5 represented a White/European American Grade III patient that does not display detectable wild-type and truncated *HMGA2* (Figure 1B). Patient tissue B10 represented a medium-scoring Grade III tissue for wild-type and truncated *HMGA2* (Figure 1C), while Patient I6 was representative of a high-scoring wild-type *HMGA2* sample from a Black/African American metastatic patient (Figure 1D). We manually counted the blue dots (wild-type *HMGA2*) and red dots (truncated *HMGA2*), and Table 1 summarizes the relative expression of wild-type and truncated *HMGA2* mRNA detected in the Fox Chase tissue microarray.

**Figure 2:**
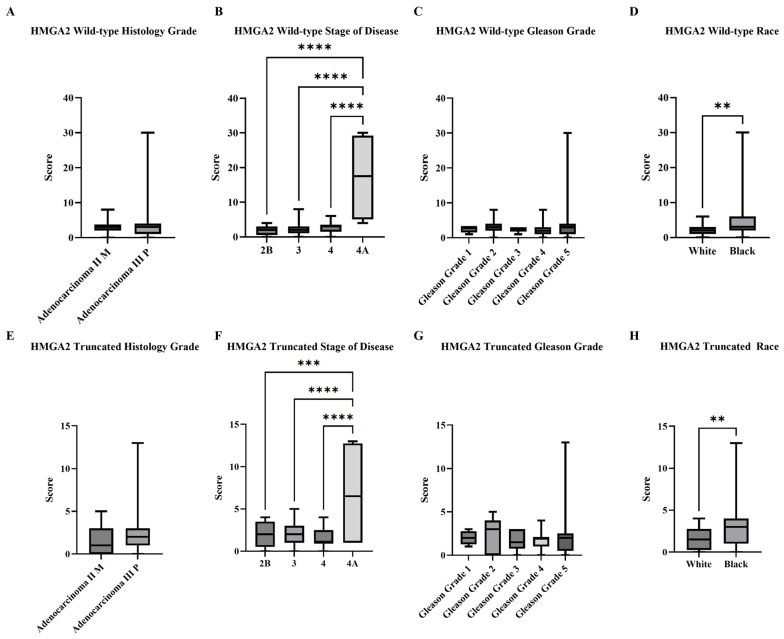
Summarized charts of Fox Chase tissue microarray based on subjective standard scoring. RISH images from Fox Chase Cancer Center tissue array that contained Black (*n* = 12) and White (*n* = 27). PCa were analyzed by manual standard scoring of wild-type and truncated *HMGA2* mRNA. Statistical comparison of wild-type *HMGA2* based on: (**A**) Histology grade; (**B**) Stage of diseases; (**C**) Gleason grade; and (**D**) Races. Comparison of truncated *HMGA2* based on: (**E**) Histology grade; (**F**) Stage of diseases; (**G**) Gleason grade; and (**H**) Races. Statistical analyses were done using GraphPad Prism software (Student’s *t*-test for (**A**,**D**,**E**,**H**); one-way ANOVA, Šídák’s multiple comparisons test for (**B**,**C**,**F**,**G**), **** *p* < 0.0001 *** *p* < 0.001, ** *p* < 0.01). Bars represent the SD of the mean.

Based on the manual subjective scoring, expression levels of wild-type and truncated *HMGA2* signals detected in tissue samples were analyzed by histological grade, disease stage, Gleason grade, and race (Black and White patients; see Figure 2). There was no statistical difference according to histological grade (Figure 2A,E); however, both isoforms showed significantly higher expression in Stage 4A compared with the other three stages (Figure 2B,F). Additionally, there was no statistical difference according to Gleason Grade (Figure 2C,G). Finally, Black patients exhibited significantly higher levels of both isoforms compared to the White patients (Figure 2D,H). This method, however, was highly subjective and required extensive time for image capture, signal comparison, dot counting, and scoring. Therefore, we pursued alternative approaches to achieve a more objective and efficient means of analysis.

### 2.2. Numerical Results of AI/ML Training/Validation Model

**Figure 3:**
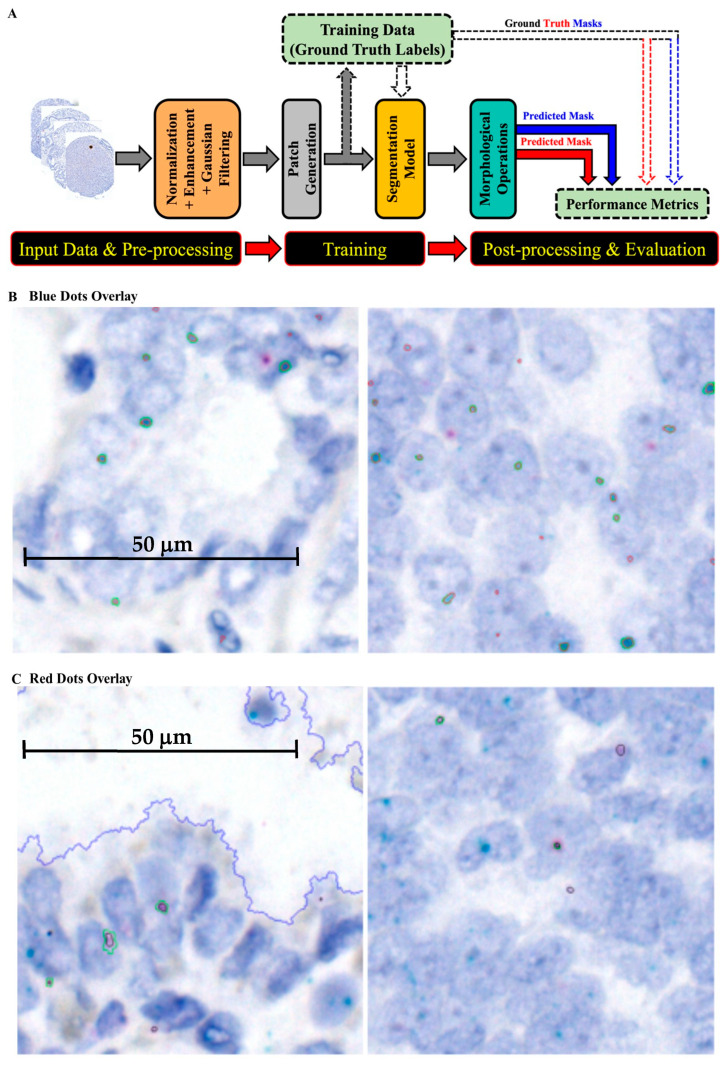
Development of the patch-based training and segmentation model for quantification of RISH signals: (**A**) Workflow of the machine learning pipeline, from data input and preprocessing through patch generation, segmentation, training, and post-processing/evaluation. The trained model was then applied to analyze RISH signals; (**B**) framework for wild-type *HMGA2* RISH image segmentation; (**C**) framework for truncated *HMGA2* RISH image segmentation.

Developing a patch-based training and segmentation model using ML modules for quantifying RISH signals is described in the Materials and Methods section and summarized in Figure 3. The multi-step AI architecture, capable of identifying and segmenting specific RNA signals, is shown in Figure 3A, and model performance in detecting blue and red dots is summarized in Table 1. To visually assess performance, predicted and ground-truth labels were overlaid on the original images (Figure 3B,C). Most steps were automated, with the exception of manual labeling, which required human expertise. Automating detection and segmentation improved accuracy, reproducibility, and efficiency of RNA signal analysis, enabling the extraction of meaningful biological insights from large-scale image datasets. This approach streamlines RISH data analysis and facilitates downstream biological interpretation.

### 2.3. AI/ML Objective Analysis of HMGA2 Isoforms in a Cohort of PCa Patient Tissue

**Figure 4:**
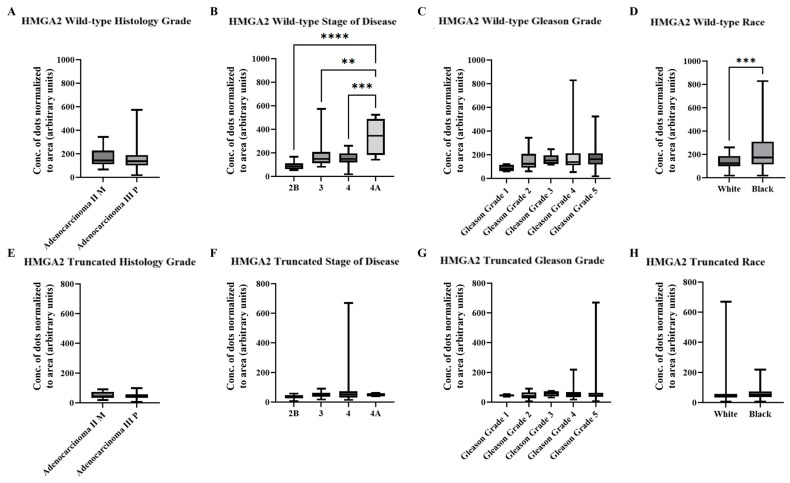
Summarized charts of Fox Chase tissue microarray using the data quantified by the trained AI. Statistical comparison of wild-type *HMGA2* based on: (**A**) Histology grade; (**B**) Stage of diseases; (**C**) Gleason grade; and (**D**) Races. Comparison of truncated *HMGA2* based on: (**E**) Histology grade; (**F**) Stage of diseases, (**G**) Gleason grade; and (**H**) Races. Statistical analyses were done using GraphPad Prism software (Student’s t-test for (**A**,**D**,**E**,**H**); one-way ANOVA, Šídák’s multiple comparisons test for (**B**,**C**,**F**,**G**), **** *p* < 0.0001, *** *p* < 0.001, ** *p* < 0.01). Bars represent the SD of the mean.

We next quantified our RISH images using the newly developed AI/ML model. Table 2 summarizes the wild-type and truncated *HMGA2* mRNA signals detected by the AI model in the Fox Chase tissue microarray. The higher signal of wild-type *HMGA2* compared to truncated *HMGA2* was consistent with the manual scoring system. Histological grade again showed no statistically significant differences for wild-type or truncated *HMGA2* (Figure 4A,E). However, wild-type *HMGA2*, but not truncated *HMGA2*, showed significantly increased expression with advancing stage (Figure 4B,F), while no statistical differences were observed by Gleason grade (Figure 4C,G). Additionally, Black patients exhibited higher levels of wild-type *HMGA2* compared to White patients, whereas no significant difference was observed in the expression of truncated *HMGA2* (Figure 4D,H).

### 2.4. AI/ML Objective Analysis of HMGA2 Isoforms in a Larger Cohort of PCa Patient Tissue

While the AI-based architecture was completing training, we conducted additional RISH experiments using a tissue microarray purchased from Tissue Array (formerly US BioMax), which includes samples from patients of Asian descent, along with three individual tissue slides from Kenyan patients to supplement the Black patient samples in the Fox Chase Cancer Center tissue microarray (Table S1 and Table 3). In total, we analyzed PCa tissue samples from 15 Black, 27 White, and 40 Asian individuals. The newly developed AI tool was then applied to quantify all resulting images. Unlike the Fox Chase Cancer Center tissues and the Kenyan patients, the Tissue Array samples showed a higher number of red signals (truncated *HMGA2*) than blue signals (wild-type *HMGA2*) in the Asian group (Table S1).

**Figure 5:**
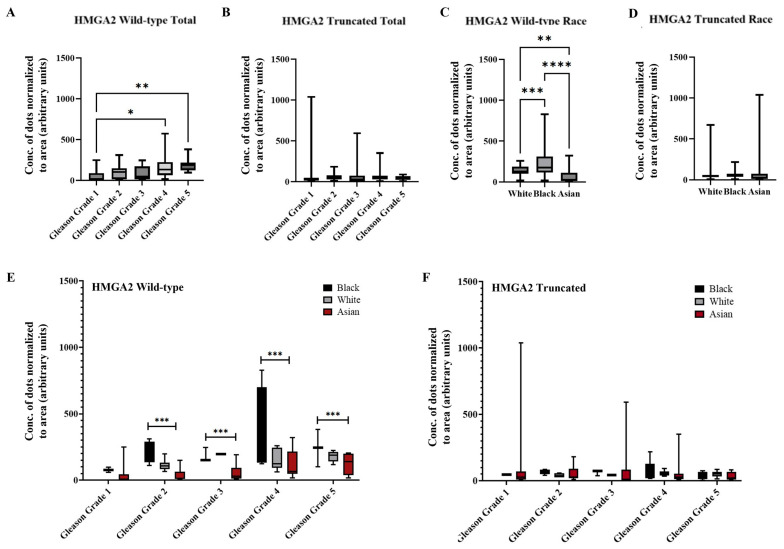
Summarized charts of patient samples of different ethnicities using the data quantified by the trained AI. In combined sources of tissue samples from White (*n* = 27), Black (*n* = 15) and Asian (*n* = 43) PCa patients, statistical comparison of (**A**) wild-type *HMGA2* and (**B**) truncated *HMGA2* based on Gleason grade. Analysis was performed according to race (White, Black or Asian) for (**C**) wild-type *HMGA2*; and (**D**) truncated *HMGA2* RNA. We compared the expression of (**E**) wild-type *HMGA2*; and (**F**) truncated *HMGA2* by Gleason grade in the three races. Statistical analyses were done using GraphPad Prism software (one-way ANOVA, Šídák’s multiple comparisons test for A, B, C, and D; two-way ANOVA, Tukey’s multiple comparisons test for (**E**,**F**), **** *p* < 0.0001, *** *p* < 0.001, ** *p* < 0.01, * *p* < 0.05). Bars represent the SD of the mean.

Based on the data from all three sample sources, analyses focused on Gleason grade and race. As shown in Figure 5A, wild-type *HMGA2* expression increased with Gleason grade, with significantly higher expression in grades 4 and 5 compared to grade 1. In contrast, truncated *HMGA2* showed no statistically significant differences across Gleason grades (Figure 5B). Additionally, wild-type *HMGA2* expression varied significantly by ethnicity, with the highest expression observed in Black patients and the lowest in Asian patients (Figure 5C). No significant differences by race were detected for the truncated isoform (Figure 5D). Figure 5E presents a combined analysis of Gleason grade and race for wild-type *HMGA2*, revealing a marked difference between Black and Asian patients in samples with Gleason grade > 2. Consistently, the truncated *HMGA2* showed no statistically significant differences in this combined analysis (Figure 5F).

## 3. Discussion

In this study, we explored two methods to assess the expression of wild-type and truncated *HMGA2* using RNA in situ hybridization (RISH). The first approach, a manual self-scoring system based on the subjective evaluation of 10 images per sample, proved to be time-consuming and visually fatiguing. Additionally, this method may miss signals present in unexamined regions of the tissue. Statistical analyses revealed consistent patterns of wild-type *HMGA2* expression across histological grade, disease stage, Gleason grade, and race when comparing results from the manual scoring system and the machine learning approach. These matched results support the feasibility of using machine learning as a substitute for manual scoring in the analysis of mRNA expression.

Our approach builds on existing image analysis methods, but it is the first to apply a machine learning-assisted pipeline for isoform-specific *HMGA2* quantification from RISH images. This integration is novel because it provides a low-cost and accessible workflow, preserves spatial information, and is applied to a racially diverse prostate cancer cohort. Previous studies have detected *HMGA2* isoforms using RT-PCR/quantitative PCR, gel electrophoresis, Sanger sequencing, or bulk RNA sequencing, all of which lack spatial resolution [21,22,23]. Immunohistochemistry can assess overall protein expression, but cannot distinguish isoforms [24], and manual RISH scoring is subjective and not scalable. By providing automated, spatially resolved isoform quantification in tissue, our AI/ML-assisted pipeline fills an important methodological gap.

However, for truncated *HMGA2*, the machine learning model did not detect statistically significant differences, whereas the subjective method indicated significance in categories such as disease stage and race. This discrepancy may stem from bias inherent in the manual scoring system, which relies heavily on visual judgment and does not account for signals outside the ten selected image fields. Furthermore, the manual scoring was constrained to a scale of 0–5, while the AI-based quantification can detect differences exceeding fivefold. Even in the positive control, truncated *HMGA2* expression was generally lower than wild-type *HMGA2* across all groups, with the exception of approximately half of the cases within the Asian group. In such low-expression cases, even one or two outliers can inflate the standard deviation, thereby reducing statistical significance.

As with most research, the strength of statistical analysis is closely tied to sample size. In this study, we analyzed a total of 85 samples, including 43 from Asian patients, 27 from European American/White patients, and 15 Black patients (12 African American and 3 from Kenya). Expanding our collaborations to include more diverse and larger cohorts would enhance the statistical power of our findings. Additionally, increasing the number of samples would benefit the machine learning model by allowing it to accumulate more training data, thereby improving accuracy and reducing potential errors. The lack of unified clinical information across different sources also limited our analysis. Specifically, the Tissue Array and Kenyan samples did not include disease stage data, a category that showed strong statistical significance in the Fox Chase array. Standardizing clinical data collection in future studies will be essential for more robust and comprehensive analyses. In addition, the Formalin-Fixed Paraffin-Embedded (FFPE) slides and the two tissue microarrays were prepared by three different institutes, which may vary in their processes for paraffin embedding, sectioning, and slide preservation.

In our analysis, higher wild-type *HMGA2* expression was associated with more advanced prostate cancer stages and higher Gleason grades. Notably, patients of African origin exhibited significantly elevated wild-type *HMGA2* expression during cancer progression, while patients of Asian origin showed lower expression levels. This observed disparity in wild-type *HMGA2* expression across ethnic groups warrants further investigation with larger and more diverse cohorts and may potentially serve as a risk assessment biomarker for PCa prevention and prognosis. While these racial and ethnic differences in wild-type *HMGA2* expression are noteworthy, they remain correlative and do not yet reveal underlying biological, environmental, or clinical contributors. This study represents an initial analytical step focused on establishing and validating an accessible AI/ML-assisted RISH pipeline. Ongoing work, including our collaboration with AC3, is aimed at expanding cohort diversity, improving clinical annotation, and conducting mechanistic and contextual analyses to better understand the drivers of *HMGA2* isoform variation across populations.

Although truncated *HMGA2* did not reach statistical significance in AI-based quantification, we observed notable expression patterns in patients of Asian descent. Specifically, while wild-type *HMGA2* signals were generally higher than truncated *HMGA2* in the Black patient samples, several Asian patients exhibited markedly higher levels of truncated *HMGA2* compared to the wild-type isoform. Due to considerable variability within this group, these differences did not reach statistical significance. Nevertheless, the presence of elevated truncated *HMGA2* in a subset of Asian patients merits further attention in future studies.

Due to the lack of antibodies that can specifically distinguish between wild-type and truncated *HMGA2*, there are currently no statistical studies directly addressing the roles of these two isoforms in health disparities. The truncated isoform of *HMGA2* has been implicated in oxidative stress, cell proliferation, and ferroptosis, while the wild-type isoform has been implicated in promoting EMT [7,10]. For example, one study showed that overexpression of truncated *HMGA2* lacking the 3′ UTR induced non-malignant in vivo expansion of hematopoietic stem cells in juvenile pigtailed macaques [25]. Another report indicated that truncated *HMGA2* expression promotes a more mesenchymal (stem-like) phenotype in an immortalized mesenchymal stem-like cell (MSC) line, suggesting that its overexpression may provide tumors with stem-like properties [9]. To our knowledge, this may be the first study examining the connection between wild-type and truncated *HMGA2* and health disparities.

The proposed AI/ML-assisted pipeline demonstrates strong feasibility for reproducible quantification of *HMGA2* isoforms in PCa tissues using RISH. Our tailored data preprocessing, Double U-Net architecture, custom probe-specific ground-truth labeling, and batch-wise validation enable highly accurate RNA-dot segmentation in challenging FFPE prostate tissues—achieving (Dice Similarity Coefficient) (DSC) scores up to 99%—and reveal biologically significant patterns across race, stage, and Gleason grade. These capabilities overcome common limitations of current methods, such as subjective interpretation and reliance on expensive software in manual or software-based approaches, providing a reproducible, accessible, and clinically meaningful framework for studying *HMGA2* isoforms and cancer disparities.

While commercial software tools have proven useful, they often require extensive user input and may not generalize well to diverse datasets or tissue types. Although accurate under expert supervision, manual methods are time-consuming, visually fatiguing, prone to observer bias, and not scalable for large datasets or longitudinal studies.

In contrast, our AI-based pipeline introduces a robust method to identify and quantify *HMGA2*, a biomarker associated with cancer metastasis and resistance therapy, as examined through RISH. The pipeline integrates advanced preprocessing techniques (contrast-limited adaptive histogram equalization (CLAHE), patch-based) with a Double U-Net-based segmentation framework to isolate RNA dots in RISH images accurately. This approach enables more accurate and consistent dot detection through pixel-level segmentation, improves scalability with patch-based training for whole-slide image analysis, reduces computational costs, and minimizes reliance on manual oversight.

Our results, evaluated using metrics such as DSC and Intersection over Union (IoU), demonstrate that the model can reliably segment and quantify RNA dots, even in challenging tissue environments with overlapping signals or variable staining intensities.

A key strength of our pipeline is its accessibility, particularly for under-resourced or low-resource settings. Because the pipeline was implemented using widely available Python libraries (e.g., TensorFlow, OpenCV), it can be deployed without the need for expensive commercial software licenses or proprietary hardware. In addition, the patch-based training approach reduces dependency on high-end GPU clusters by enabling training on mid-tier hardware, such as the Dell Precision with RTX A5000 used for this study. This makes the approach more accessible for smaller research institutions and clinical labs. Moreover, using standardized preprocessing (e.g., CLAHE for contrast enhancement and Gaussian filtering for noise reduction) enhances generalizability across tissue types and imaging conditions.

The modular design of our AI pipeline enables flexible and collaborative use across research centers. Each component, including preprocessing, segmentation, and evaluation, can be independently modified or extended, making the pipeline adaptable to varying institutional needs and research goals. Similarly, standardized ground truth patch datasets can be curated and shared to support federated learning or domain adaptation, allowing collaborative model development without centralizing sensitive data. This is particularly valuable for studying population-level differences, such as racial disparities in *HMGA2* isoform expression, by ensuring that models are trained on diverse, representative data. The pipeline’s robustness promotes cross-institutional collaboration, even among institutions with differing levels of computational resources, facilitating data sharing, model transferability, and equitable AI development without compromising performance.

To facilitate comparison with prior work, Table 4 summarizes the proposed method alongside representative RNA spot detection and segmentation approaches reported in the literature. It is important to note that direct numerical comparison across methods is limited by differences in problem formulation and evaluation metrics, including object-detection-based measures (e.g., average precision), signal similarity metrics (e.g., Pearson correlation), and pixel-wise segmentation metrics (e.g., Dice coefficient). Consequently, the reported performance values should be interpreted as contextual rather than strictly quantitative comparisons. Despite these differences, the proposed method demonstrates high segmentation performance. However, the approach requires generating the ground truth masks for supervised training, employs a 64 × 64 patch-based training that may miss larger spatial patterns, and its generalizability needs future external validation, as evaluation was performed exclusively on in-house RNA ISH slides and may require stain normalization for broader deployment.

## 4. Materials and Methods

### 4.1. Patient Cohort and Sample Collection

Formalin-fixed, paraffin-embedded (FFPE) PCa tissue microarrays comprising 85 prostate adenocarcinoma samples were utilized in this study. Tissue samples were retrospectively obtained from Tissue Array (Cat PR821, Derwood, MD, USA), Kenyatta National Hospital and MP Shah Hospital in Kenya, and Fox Chase Cancer Center. These sites are part of the African-Caribbean Cancer Consortium (AC3), an international collaborative network of researchers aimed at studying cancer incidence, progression, and molecular characteristics in populations of African and Caribbean descent.

All procedures complied with relevant laws and institutional guidelines and were approved by the appropriate institutional committee(s). The samples included both metastatic and non-metastatic adenocarcinoma. Clinical information for the cohort included Gleason score, patient age, and race (Table 1, Table 2, Table 3 and Table S1). Patient ethnicity was represented as follows: African American (*n* = 12), European American (*n* = 27), Kenyan (*n* = 3), and Asian (*n* = 40). Gleason scores ranged from 4 to 10, and patient ages ranged from 51 to 81 years.

### 4.2. RNA In Situ Hybridization (RISH) Probe Design

**Figure 6:**
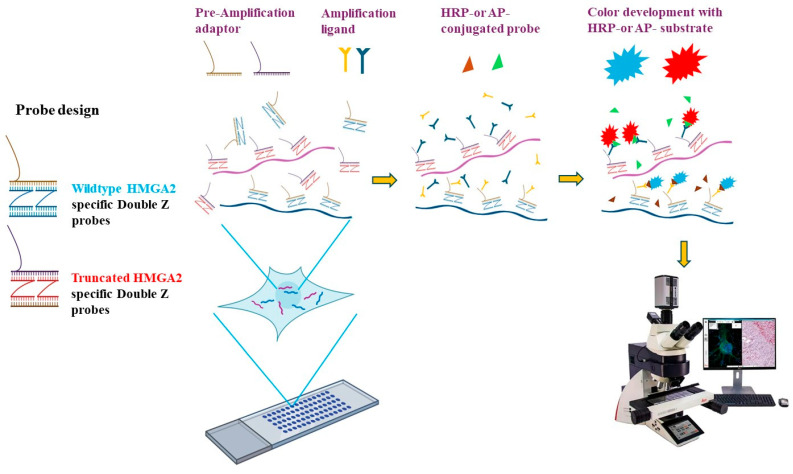
Principle of RNA in situ hybridization (RISH). Probes were designed in the double Z configuration to ensure specificity, based on the different exons spliced in the wild-type *HMGA2* (exon 5) and truncated *HMGA2* (exon 4). The upper sequence of the probe is complementary to the pre-amplification adaptor (Pre-Amp). After binding to RNA from the pre-treated FFPE slides, amplification ligands attach to the probe-pre-Amp complex. A horseradish peroxidase (HRP)- or alkaline phosphatase (AP)-conjugated probe then binds to the amplification ligands, enabling the detection of colorful signals in the presence of the HRP or AP substrates. RISH images were collected using the Aperio VERSA digital scanner (Leica Biosystems, Inc., Deer Park, IL, USA).

Unlike Northern blotting and quantitative PCR, RNA RISH enables visualization and localization of target mRNA transcripts within cells and tissue samples. In cases where specific antibodies are unavailable to distinguish between isoforms of the same gene, RISH provides a powerful approach for studying isoform expression of disease-related genes in tissue sections. The wild-type *HMGA2* isoform includes exon 5 and a long 3′ untranslated region (L-3′UTR), whereas the truncated isoform replaces this region with exon 4 and a short 3′UTR (S-3′UTR). To detect these isoforms, specific probes were designed to target exon 5 for the wild-type isoform and exon 4 for the truncated isoform of *HMGA2*. The probes consist of short primers in a double Z configuration paired with pre-amplification adaptors, forming the specific RISH probes (Figure 6).

### 4.3. RISH Assay

RISH was performed using the ACD RNAscope 2.5 Duplex Detection kit (Bio-Techne, Minneapolis, MN, USA) according to the manufacturer’s instructions. An outline of the assay is shown in Figure 6, where formalin-fixed, paraffin-embedded (FFPE) tissue sections underwent a series of preparatory steps, including deparaffinization, permeabilization, antigen retrieval, and protein removal, before hybridization with the designed probes. Following multiple amplification steps, probes conjugated with horseradish peroxidase (HRP) or alkaline phosphatase (AP) enable signal detection. Color development was achieved by applying specific substrates for HRP or AP, allowing visualization of the target isoforms (Figure 6). More specifically, tissue microarray and FFPE slides were baked at 60 °C for 30 min. Slides were deparaffinized with xylene, followed by ethanol. After treatment with hydrogen peroxide for 10 min, slides were boiled in target retrieval solution for 15 min and then treated with protease for 30 min. Following brief washes, slides were incubated with probes specific to wild-type *HMGA2* and truncated *HMGA2* for 2 h at 40 °C. Slides then underwent a series of amplification steps, followed by color development. Slides were subsequently imaged using the Aperio Scanner, Leica Biosystems, Inc., Deer Park, IL, USA.

### 4.4. RISH Analysis Using Subjective Scoring Methods

We analyzed the number of blue dots (*HMGA2*-WT) or red dots (*HMGA2*-TR) in tumor tissue by visual counting of the RISH images. Red dots represent truncated *HMGA2* transcripts, which were detected using a probe conjugated to alkaline phosphatase (AP), and blue dots represent wild-type *HMGA2* transcripts, detected using a probe conjugated to horseradish peroxidase (HRP). For each circular tissue sample, ten images were captured at 40× magnification (one at the center and nine around the periphery). The expression score for each image (ranging from 0 to 5) was determined by comparing the signal intensity to the HeLa control. HeLa cells were utilized as a positive control, assigning them a score of 5 for wild-type (more than 80 blue dots/field) and truncated (more than 40 red dots/field). Score 4 is 60–80 blue dots/30–40 red dots, score 3 is 40–60 blue dots/20–30 red dots, score 2 is 20–40 blue dots/10–20 red dots, score 1 is 0–20 blue dots/0–10 red dots and score 0 is neither blue nor red dot was observed in the field.

### 4.5. AI/ML Pipeline for RISH Analysis

To analyze RISH images, we developed a multi-step AI-based architecture capable of identifying and segmenting specific RNA signals. The pipeline integrates learnable modules with state-of-the-art segmentation techniques to address the unique challenges of RISH image analysis. Segmentation plays a central role in processing RISH images: by dividing each image into distinct regions corresponding to RNA signals (visualized as blue and red dots in the pathology images) and background, it ensures that subsequent analysis focuses on biologically relevant features. Effective segmentation is essential for improving the accuracy of RNA signal detection, reducing noise, and enabling downstream quantification of gene expression [29]. In addition, segmentation allows researchers to visualize and interpret spatial patterns of RNA distribution within tissue sections, providing deeper insights into biological processes. The following section details the sequential analysis steps of the developed pipeline.

### 4.6. Preprocessing Stage

Data preprocessing is an essential step for RISH analysis, not only to ensure the generalizability of the proposed model but also to enhance image quality and improve the reliability of downstream quantitative analysis. First, all RISH images were converted to a standardized format and normalized by scaling pixel intensity levels to the range [0, 1] using min–max normalization, which reduces variability introduced by differences in acquisition conditions. To enhance local contrast and better visualize RNA signal dots, we applied the contrast-limited adaptive histogram equalization (CLAHE) technique [24] to each color channel using a clip limit of 2.0 and a tile grid size of 8 × 8 pixels. This step improves the visibility of fine structures, particularly the blue and red chromogenic dots that represent RNA expression. To further suppress high-frequency noise while preserving dot-like structures, a Gaussian filter with a kernel size of 3 × 3 and standard deviation 1.0 was applied, making the RNA dots easier to detect.

Because training directly on whole-slide images is computationally expensive and memory-intensive, a patch-based training strategy was adopted. Each preprocessed RISH image was divided into non-overlapping patches of 64 × 64 pixels, a size selected to balance contextual information by capturing fine-grained details with manageable computational load. For ground truth generation, blue and red RNA signal dots within each patch were manually traced and segmented using a graphics tablet and selection tools, then reviewed and validated by an experienced domain expert. Pixels corresponding to RNA signal dots were labeled as foreground (value 1, White), and all remaining regions were labeled as background (value 0, Black), resulting in binary segmentation masks aligned pixel-wise with the original patches. These patch–mask pairs formed the supervised training and evaluation dataset for the segmentation model.

### 4.7. Model Training and Experimental Settings

Segmentation was performed using the Double U-Net architecture, which is well-suited for handling complex biological images due to its ability to operate across multiple scales [30]. This model extends the traditional U-Net by stacking two U-Net models sequentially, thereby enhancing feature extraction and segmentation precision. The first U-Net captures coarse-grained features, while the second refines them to improve segmentation accuracy. Both U-Nets consist of encoder–decoder pathways with skip connections, ensuring the preservation of spatial details across scales and facilitating feature reuse. Each encoder block consists of convolutional layers, batch normalization, and Rectified Linear Unit (ReLU) activation, followed by max-pooling for down-sampling. In contrast, each decoder block uses transposed convolutions for up-sampling. The model leverages a dice loss function for training, which optimizes overlap between predicted and ground truth segmentations, making it highly suitable for pixel-level biomedical image segmentation. This architecture is particularly effective for capturing multi-scale features and resolving complex structures, making it effective for segmenting RNA signals (red and blue dots) in ISH images. For training, each image and its corresponding label were divided into 64 × 64-pixel patches. Smaller patch sizes reduce computational complexity while balancing localized detail with sufficient contextual information, which is crucial for accurately segmenting fine features such as RNA dots in ISH images. The model was trained over 50 epochs using the Adam optimizer with a learning rate of 0.001 and the dice loss function. ReLU activations were applied within the convolutional layers to introduce non-linearity, and a final sigmoid activation was used in the output layer to generate pixel-wise probabilities for binary classification. The dataset was randomly split into 4437 image patches (80%) for training and 493 patches (20%) for testing. The proposed architecture was implemented in Python 3.0 using TensorFlow/Keras 2.15.0, NumPy 1.26.4, OpenCV 4.5.5, and Matplotlib 3.8.0. All experiments were conducted on a Dell Precision 3650 Tower workstation (Intel Core (TM) eight-core CPU running at 2.50 GHz, 64 GB RAM, and with NVIDIA RTX A500 (NVIDIA, Santa Clara, CA, USA)), running Windows and executed in Jupyter Notebook 7.0.8.

### 4.8. Post-Processing and Evaluation

After training, we tested the model on new image patches, where it generated predicted segmentations of the RNA dots. To refine these predictions, we applied morphological operations such as dilation and erosion to reduce noise and ensure a cleaner, more continuous representation of segmented RNA dots. All segmented patches were then stitched together to form a predicted mask of the whole test image, which was compared against the ground truth mask using standard evaluation metrics:

▪ Dice Similarity Coefficient (DSC): Measures the overlap between the model’s predictions and the ground truth labels.
▪ Intersection over Union (IoU): Assesses the agreement between predicted and actual RNA dots.
▪ Precision and Recall: Evaluate how many RNA dots were correctly identified versus those missed (false negatives) or incorrectly marked (false positives).

### 4.9. Statistical Analysis

Statistical analyses were performed using GraphPad Prism software version 10.6.1 (GraphPad Software, San Diego, CA, USA). Analysis of variance (ANOVA) was employed to assess differences among groups, using one-way ANOVA, Šídák’s multiple comparisons test. The significance level of *p* < 0.05 was considered statistically significant.

## 5. Conclusions

Our results suggest that higher wild-type *HMGA2* mRNA expression is associated with more advanced stages of PCa and higher Gleason grades, indicating its potential as a biomarker for risk assessment, prevention, and prognosis. We also evaluated the utility of machine learning for this type of image analysis, which may become increasingly valuable in future research. The limits of a single-disciplinary focus constrain many studies and may create bottlenecks as a result. Interdisciplinary collaboration often enables breakthroughs by combining different areas of expertise. This study serves as a strong example of such collaboration between biology and electrical engineering, and we are pleased to present our findings, which have important implications for both cancer research and technological development in engineering.

## Figures and Tables

**Table 1:** Summary of wild-type and truncated *HMGA2* for Fox Chase slide using the subjective method.

TMA Spot	Case ID *	Specimen ID	Race	Disease Stage	Metastatic	Year	Histology Grade	Gleason Pattern	Subjective Scoring *HMGA2* WT (1–40)	Subjective Scoring *HMGA2* TR (1–40)
	Pos Ctrl	21-4309							17	26
	Pos Ctrl	21-4310							4	4
	Pos Ctrl	21-4312							30	30
	Pos Ctrl	21-4316							40	40
B2	40	1012050	White	2B	Non-Meta	2011	Adenocarcinoma III P	034	4	4
B5	41	1012216	White	2B	Non-Meta	2011	Adenocarcinoma III P	033	3	3
B6	42	1012220	White	2B	Non-Meta	2011	Adenocarcinoma III P	034	2	4
B9	43	1012335	White	2B	Non-Meta	2011	Adenocarcinoma III P	034	3	3
B10	44	1012402	White	2B	Non-Meta	2011	Adenocarcinoma III P	034	2	4
C6	47	1013484	White	4		2012	Adenocarcinoma III P	045	5	4
C9	48	1014164	Black	3	Non-Meta	2013	Adenocarcinoma III P	044	4	4
D2	50	1015479	Black	3		2014	Adenocarcinoma II M	034	6	3
D5	51	1015775	Black	3		2014	Adenocarcinoma	034	3	3
D6	52	1015805	Black	3	Non-Meta	2014	Adenocarcinoma II M	034	8	5
D8	53	1015945	Black	3		2014	Adenocarcinoma III P	044	3	3
D10	54	1017035	White	4		2015	Adenocarcinoma III P	044	4	1
E5	56	1017270	White	4		2015	Adenocarcinoma III P	044	5	3
E6	57	1017534	White	4		2015	Adenocarcinoma III P	044	3	3
E9	58	1017541	Black	3	Non-Meta	2015	Adenocarcinoma III P	043	3	3
F5	61	1017903	White	4		2016	Adenocarcinoma III P	034	3	3
G3	65	1019042	White	4		2017	Adenocarcinoma III P	054	4	2
G4	66	1019132	White	4	C0	2017	Adenocarcinoma III P	045	3	3
H2	70	1019428	White	4		2017	Adenocarcinoma III P	054	6	2
H6	72	1019693	White	3	c0	2017	Adenocarcinoma III P	045	4	2
I3	75	1020025	White	4	c1B	2017	Adenocarcinoma III P	045	4	1
I6	77	1020766	Black	4A	p1	2018	Adenocarcinoma III P	045	30	13
I9	78	1022418	Black	4A	p1	2020	Adenocarcinoma III P	053	7	1

* Total 39 samples, 27 White and 12 Black. (Set positive control as score 4 for 10 views: highest: 40; WT score ≥ 4, or TR score ≥ 3, 23 patients with positive score.)

**Table 2:** Summary of wild-type and truncated *HMGA2* for Fox Chase slide using a trained machine learning model.

TMA Spot	Case ID *	Specimen ID	Race	Stage	Metastatic	Year	Histology Grade	Gleason Pattern	Wild-Type *HMGA2*	Truncated *HMGA2*
B3	40	1012050	White	2B	Non-Meta	2011	Adenocarcinoma III P	034	166.64	40.25
B4	41	1012216	White	2B	Non-Meta	2011	Adenocarcinoma III P	033	57.64	46.17
B7	42	1012220	White	2B	Non-Meta	2011	Adenocarcinoma III P	034	125.79	18.90
B9	43	1012335	White	2B	Non-Meta	2011	Adenocarcinoma III P	034	84.87	41.67
B10	44	1012402	White	2B	Non-Meta	2011	Adenocarcinoma III P	034	101.82	57.47
C2	45	1012441	White	2B	Non-Meta	2011	Adenocarcinoma II M	033	97.59	44.32
C5	46	1012914	White	2B	Non-Meta	2011	Adenocarcinoma III P	044	61.33	40.12
C8	48	1014164	Black	3	Non-Meta	2013	Adenocarcinoma III P	044	125.15	17.73
C10	49	1014392	Black	3		2013	Adenocarcinoma III P	045	100.00	36.82
D3	50	1015479	Black	3		2014	Adenocarcinoma II M	034	236.45	65.45
D6	52	1015805	Black	3	Non-Meta	2014	Adenocarcinoma II M	034	310.18	85.89
D8	53	1015945	Black	3		2014	Adenocarcinoma III P	044	572.71	29.49
E8	58	1017541	Black	3	Non-Meta	2015	Adenocarcinoma III P	043	148.25	73.65
F2	60	1017845	Black	3	Non-Meta	2016	Adenocarcinoma II M	043	151.03	38.03
F6	62	1018076	Black	3	Non-Meta	2016	Adenocarcinoma II M	034	111.02	76.47
F10	64	1018715	White	3	C0	2016	Adenocarcinoma III P	055	161.40	56.31
G9	68	1019179	White	3	c0	2017	Adenocarcinoma III P	044	116.58	57.76
G10	69	1019424	White	3	c0	2017	Adenocarcinoma III P	044	150.33	33.24
H4	71	1019525	White	3	c0	2017	Adenocarcinoma III P	045	123.62	36.36
H6	72	1019693	White	3	c0	2017	Adenocarcinoma III P	045	215.96	51.40
H11	74	1019931	White	3	c0	2017	Adenocarcinoma II M	034	135.55	31.80
C6	47	1013484	White	4		2012	Adenocarcinoma III P	045	190.05	54.83
D11	54	1017035	White	4		2015	Adenocarcinoma III P	044	148.89	48.22
E2	55	1017126	White	4		2015	Adenocarcinoma II M	034	111.47	44.46
E4	56	1017270	White	4		2015	Adenocarcinoma III P	044	245.57	71.05
E6	57	1017534	White	4		2015	Adenocarcinoma III P	044	259.68	91.99
E11	59	1017573	White	4		2015	Adenocarcinoma III P	044	120.11	26.39
F4	61	1017903	White	4		2016	Adenocarcinoma III P	034	197.77	58.38
F8	63	1018329	White	4	p1A	2016	Adenocarcinoma III P	045	118.17	78.32
G2	65	1019042	White	4		2017	Adenocarcinoma III P	054	193.60	40.00
G4	66	1019132	White	4	C0	2017	Adenocarcinoma III P	045	212.07	87.14
G6	67	1019161	White	4		2017	Adenocarcinoma III P	044	122.64	70.32
H3	70	1019428	White	4		2017	Adenocarcinoma III P	054	257.86	63.63
H8	73	1019800	White	4		2017	Adenocarcinoma II M	043	195.24	42.96
I2	75	1020025	White	4	c1B	2017	Adenocarcinoma III P	045	188.67	14.54
I4	76	1020541	Black	4	p1	2018	Adenocarcinoma III P	053	179.88	20.00
I6	77	1020766	Black	4A	p1	2018	Adenocarcinoma III P	045	523.59	62.86
I9	78	1022418	Black	4A	p1	2020	Adenocarcinoma III P	053	307.92	52.06

* Total 39 samples, 27 White and 12 Black. (Set positive control as score 4 for 10 views: highest: 40; WT score ≥ 4, or TR score ≥ 3.)

**Table 3:** Summary for Kenyan Cases with higher wild-type and truncated *HMGA2* using a trained machine learning model.

No.	Age	PSA	Year of Diagnosis	Gleason Score	MRI	Bone Scan	Blue Spots *HMGA2* WT	Red Spots *HMGA2* TR
1	64	41	2022	5 + 4	Negative	Negative	19.15	5.245
3	63	80	2022	4 + 3	Positive	Not done	246.1603448	74.11603448
20	73	193	2021	4 + 4	Not done	Not done	829.0873913	218.5

**Table 4:** Comparison of Performance and Features of AI/ML Pipeline versus Existing Methods.

Method	Method	Accuracy/Performance	Computational Complexity	Limitations
StarDist [26]	CNN object detection	Very high for well-separated RNA dots, Average precision 86.41%	Fast inference; lightweight	Fails on tiny granular ISH dots; shape-biased
DeepSpot [27]	CNN enhancement → thresholding	Improves spot SNR by ~30%. Pearson 76.40%	Lightweight	Not a full segmentation model
SpotLearn [28]	CNN dot detection	High accuracy in dense tissues	Medium	Needs large annotated data
Proposed	U-Net segmentation of RNA dots	High accuracy is detecting RNA dots. DC 99.2% and 99.0% for blue and red dots simultaneously.	Medium	manual annotation for ground truth and external validation is required

## Data Availability

The data presented in this study are available upon reasonable request from the corresponding author. For reproducibility, the full analysis code, including custom modifications, will be made publicly available via a GitHub 1.0 online repository upon publication.

## Supplementary Materials

The following supporting information can be downloaded at: https://www.mdpi.com/article/10.3390/ijms27010196/s1.

## Abbreviations

The following abbreviations are used in this manuscript:

AI	artificial intelligence
AI/ML	artificial intelligence and machine learning
ANOVA	analysis of variance
AP	alkaline phosphatase
AR	androgen receptor
CEAMLS	Center for Equitable AI and Machine Learning Systems
CLAHE	contrast-limited adaptive histogram equal ization
CNNs	convolutional neural networks
DSC	dice similarity coefficient
DL	deep learning
EMT	epithelial–mesenchymal transition
FFPE	formalin-fixed paraffin-embedded
*HMGA2*	High Mobility Group AT-Hook 2
*HMGA2*-WT	High Mobility Group AT-Hook 2-wild-type
*HMGA2*-TR	High Mobility Group AT-Hook 2-truncated
HRP	horseradish peroxidase
IOU	Intersection over Union
L-3′UTR	long 3′ untranslated region
ML	machine learning
PCa	prostate cancer
ReLU	rectified linear unit
RISH	RNA in situ hybridization
RT-PCR	reverse transcription polymerase chain reaction
S-3′UTR	short 3′untranslated region
TMAs	tissue microarrays
UTR	3′ untranslated region
