# Supplementary material for: AI/ML-Assisted Detection of HMGA2 RNA Isoforms in Prostate Cancer Patient Tissue
Source: Int J Mol Sci. 2025 Dec 24;27(1):196. doi: 10.3390/ijms27010196 (PMC12785557; doi:10.3390/ijms27010196)
Supplement: Supplementary file 1 [file ijms-27-00196-s001.zip › ijms-4030536-supplementary.pdf]

**Table S1.** Summary of wild-type and truncated *HMGA2* for Tissue Array.com slide using trained Machine learning model.

| **Pos.** | **Age** | **Site** | **Pathology diagnosis** | **TNM** | **Grade** | **Type** | **Gleason Score** | **Gleason Grade** | **Blue spots *HMGA2* WT** | **Red spots *HMGA2* TR** |
|---|---|---|---|---|---|---|---|---|---|---|
| A1 | 79 | Prostate | Adenocarcinoma | T2N0M0 | 1 | Malignant | 2+4 | 1 | 1.69 | 9.95 |
| A2 | 77 | Prostate | Adenocarcinoma | T2N0M0 | 1 | Malignant | 2+3 | 1 | 1.02 | 9.37 |
| A3 | 72 | Prostate | Adenocarcinoma | T2N0M0 | 1 | Malignant | 2+4 | 1 | 1.65 | 36.32 |
| A4 | 78 | Prostate | Adenocarcinoma | T2N0M0 | 1 | Malignant | 3+3 | 1 | 13.29 | 41.61 |
| A5 | 64 | Prostate | Adenocarcinoma | T2N0M0 | 1 | Malignant | 2+3 | 1 | 0.74 | 10.09 |
| A6 | 66 | Prostate | Adenocarcinoma | T2N0M0 | 1 | Malignant | 3+2 | 1 | 0.81 | 35.38 |
| A7 | 71 | Prostate | Adenocarcinoma | - | 1 | Malignant | 2+3 | 1 | 248.42 | 21.02 |
| A8 | 71 | Prostate | Adenocarcinoma | T2N0M0 | 2 | Malignant | 2+4 | 1 | 14.95 | 154.69 |
| A9 | 72 | Prostate | Adenocarcinoma | T2N0M0 | 2 | Malignant | 3+4 | 2 | 12.53 | 182 |
| A10 | 67 | Prostate | Adenocarcinoma | T3N1M0 | 2 | Malignant | 2+3 | 1 | 113.22 | 1037.21 |
| B1 | - | Prostate | Adenocarcinoma | T2N1M0 | 2 | Malignant | 3+4 | 2 | 9.09 | 9.47 |
| B2 | 62 | Prostate | Adenocarcinoma | T2N0M0 | 2 | Malignant | 3+4 | 2 | 8.46 | 18.26 |
| B3 | 57 | Prostate | Adenocarcinoma | T2N0M0 | 2 | Malignant | 3+4 | 2 | 149.28 | 17.13 |
| B4 | 77 | Prostate | Adenocarcinoma | T2N0M0 | 2 | Malignant | 3+4 | 2 | 107.71 | 27.26 |
| B5 | 80 | Prostate | Adenocarcinoma | T2N0M0 | 2 | Malignant | 3+4 | 2 | 20.59 | 90.46 |
| B6 | 73 | Prostate | Adenocarcinoma | T2aN0M0 | 2 | Malignant | 3+4 | 2 | 8.16 | 18.4 |
| B7 | 71 | Prostate | Adenocarcinoma | T2aN0M0 | 2 | Malignant | 3+4 | 2 | 17.24 | 88.07 |
| B8 | 75 | Prostate | Adenocarcinoma | T3N0M0 | 2 | Malignant | 3+4 | 2 | 4.76 | 68.52 |
| B9 | 73 | Prostate | Adenocarcinoma | T2N0M0 | 2 | Malignant | 4+3 | 3 | 7.99 | 137.65 |
| B10 | 66 | Prostate | Adenocarcinoma | T2N0M0 | 2 | Malignant | 4+3 | 3 | 193.35 | 591.61 |
| C1 | 61 | Prostate | Adenocarcinoma | T2N0M0 | 2 | Malignant | 4+3 | 3 | 29.88 | 11.53 |
| C2 | - | Prostate | Adenocarcinoma | T2aN0M0 | 2 | Malignant | 4+3 | 3 | 37.89 | 4.9 |
| C3 | 66 | Prostate | Adenocarcinoma | T3N0M0 | 2 | Malignant | 4+3 | 3 | 141.34 | 7.99 |
| C4 | 64 | Prostate | Adenocarcinoma | T3aN0M0 | 2 | Malignant | 4+3 | 3 | 12.86 | 4.94 |
| C5 | 60 | Prostate | Adenocarcinoma | T2N0M0 | 2 | Malignant | 4+3 | 3 | 9.66 | 10.52 |
| C6 | 81 | Prostate | Adenocarcinoma | T2N0M0 | 2--3 | Malignant | 4+3 | 3 | 45.54 | 27.8 |
| C7 | 69 | Prostate | Adenocarcinoma | T3N0M0 | 2--3 | Malignant | 4+3 | 3 | 13.74 | 15.16 |
| C8 | 80 | Prostate | Adenocarcinoma (sparse) | T3N1M0 | 1 | Malignant | 2+2 | 1 | 20.78 | 16.75 |
| C9 | 73 | Prostate | Adenocarcinoma | T2N0M0 | 2 | Malignant | 4+4 | 4 | 215.05 | 51.77 |
| C10 | 76 | Prostate | Adenocarcinoma | T2N0M0 | 2 | Malignant | 4+4 | 4 | 61.22 | 349.22 |
| D1 | 75 | Prostate | Adenocarcinoma | T2N0M0 | 2 | Malignant | 5+3 | 4 | 18.36 | 7.37 |
| D2 | 66 | Prostate | Adenocarcinoma | T4N0M0 | 2 | Malignant | 4+4 | 4 | 193.45 | 11.85 |
| D3 | - | Prostate | Adenocarcinoma | T2N1M0 | 2 | Malignant | 4+4 | 4 | 65.67 | 29.38 |
| D4 | 76 | Prostate | Adenocarcinoma | T3N0M0 | 2 | Malignant | 4+4 | 4 | 320.67 | 25.4 |
| D5 | 59 | Prostate | Adenocarcinoma | T2N0M0 | 2 | Malignant | 4+4 | 4 | 46.85 | 44.12 |
| D6 | 56 | Prostate | Adenocarcinoma | T3N0M0 | 3 | Malignant | 5+4 | 5 | 184.69 | 16.59 |
| D7 | 60 | Prostate | Adenocarcinoma | T4N0M0 | 3 | Malignant | 5+4 | 5 | 203.43 | 23.46 |
| D8 | 62 | Prostate | Adenocarcinoma | T2N0M0 | 3 | Malignant | 5+5 | 5 | 94.42 | 82.36 |
| D9 | 61 | Rib | Metastatic prostate adenocarcinoma of the right fourth rib | - | 2 | Metastasis | - | - | 45.51 | 75.03 |
| D10 | 51 | Lymph node | Metastatic prostate adenocarcinoma | - | 3 | Metastasis | - | - | 105.66 | 74.76 |
